# Meat and Men

**Published:** 1870-09

**Authors:** 


### MEAT AND MEN.

Men may live long and in health, who never taste meat, but they never can excel in anything which requires energy. The nations which eat no meat, as to the masses, are always inefficient or degraded. The hundreds of millions of Japan and China have failed in the centuries of the past in all that makes a nation or an individual grand in conception, or magnificent in accomplishment. They are to-day what they were ages ago, and they live mainly on rice and other vegetables.

The most of the Irish, at home, live chiefly on potatoes, and multitudes who reach our shores are but a short remove from idiots; they have brute force, but are mentally deficient in all that elevates.

The German laborer drinks wine, and revels in soups every day of his life, and yet it requires five of them to do as much hard work in a day as a meat-eating Yankee; indeed, when they come to this country, it requires a month's good feeding to fit them for day laboring.

Greenlanders live on oil and fat; the millions of tropical regions subsist mainly on fruit, and the hordes of Africa and Indian jungles depend on insects and nuts, and unsubstantial diet.

But the people of the world who live mainly on meats, and whose civilization commands the varieties of all climes, these are they who take the lead in all human enterprises which secure results at once magnificent and astounding in physical accomplishments and mental acquirements. The railroads that cross continents, the telegraphic wires which span the seas and girt the world, the glasses which carry us to the sun and map out the surface of the planets, the calculations which weigh the worlds of space and measure their journeyings through immensity; these be but a part of the works of, not all men, but of that portion of mankind which feeds upon the cattle of a thousand hills, upon the fishes of all seas, and the fruits and grains and products of all climes, as coming from the hand of Him who giveth us all things richly to enjoy.

